# 抗体偶联药物在非小细胞肺癌一线治疗耐药后的应用进展

**DOI:** 10.3779/j.issn.1009-3419.2025.101.14

**Published:** 2025-09-20

**Authors:** Honglin LI, Yawan JING, Jiayi SUN, Jing XU, Yalun LI

**Affiliations:** ^1^610041 成都，四川大学华西医院呼吸与危重症医学科，呼吸和共病全国重点实验室，呼吸健康研究所，疾病分子网络前沿科学中心，精准医学中心/精准医学四川省重点实验室; ^1^Department of Pulmonary and Critical Care Medicine, State Key Laboratory of Respiratory Health and Multimorbidity, Institute of Respiratory Health and Multimorbidity, Institute of Respiratory Health, Frontiers Science Center for Disease-related Molecular Net work, Precision Medicine Center/Precision Medicine Key Laboratory of Sichuan Province, West China Hospital, Sichuan University, Chengdu 610041, China; ^2^850000 拉萨，西藏自治区人民医院老年 病科; ^2^Department of Gerontology and Geriatrics, Tibet Autonomous Region People’s Hospital, Lhasa 850000, China; ^3^610041 成都，四川大学华西医院全程管理中心; ^3^Integrated Care Management center, West China Hospital, Sichuan University, Chengdu 610041, China; ^4^610041 成都，四川大学华西医院肺癌中心; ^4^Lung Cancer Center/Lung Cancer Institute, West China Hospital, Sichuan University, Chengdu 610041, China

**Keywords:** 肺肿瘤, 抗体偶联药物, 耐药性, 应对策略, Lung neoplasms, Antibody-drug conjugate, Drug resistance, Coping strategies

## Abstract

抗体偶联药物（antibody-drug conjugates, ADC）作为一类新型抗肿瘤药物，将肿瘤特异性靶向与强效细胞毒作用相结合。近年来，ADC在非小细胞肺癌（non-small cell lung cancer, NSCLC）治疗领域取得重要进展，尤其在一线治疗失败或出现耐药后的治疗序列中显示出突出的临床价值。本文拟系统综述NSCLC中常见治疗靶点及其代表性ADC的疗效与安全性证据，并进一步讨论ADC在分子结构优化、毒性管理、生物标志物筛选以及联合治疗策略等方面的进展与面临的挑战，旨在为临床实践与后续研究提供全面的理论依据与参考。

## Abstract (en)

Antibody-drug conjugate (ADC), a novel class of antineoplastic agents, combines tumor-specific targeting with potent cytotoxic activity. In recent years, ADC has achieved notable advances in the treatment of non-small cell lung cancer (NSCLC), particularly within therapeutic sequencing after failure of first-line therapy or the emergence of resistance. This paper will systematically review the efficacy and safety evidence of representative ADC in NSCLC, and further to discuss progress and challenges in ADC structural optimization, toxicity management, biomarker identification, and combination strategies, aiming to provide a comprehensive theoretical foundation and practical reference for clinical practice and future research.

晚期非小细胞肺癌（non-small cell lung cancer, NSCLC）患者预后不容乐观，IV期患者5年生存率约为9%，且多数IV期NSCLC患者最终因耐药导致疾病进展或复发^[1]^。对于晚期NSCLC，目前一线治疗主要依据分子分型和程序性死亡配体1（programmed death-ligand 1, PD-L1）表达水平进行个体化选择。目前主要包括三大类：靶向治疗、免疫联合化疗和免疫单药治疗。对于驱动基因突变阳性患者，首选靶向治疗，其中表皮生长因子受体（epidermal growth factor receptor, *EGFR*）突变患者使用第三代EGFR-酪氨酸激酶抑制剂（EGFR-tyrosine kinase inhibitors, EGFR-TKIs），如奥希替尼中位无进展生存期（median progression-free survival, mPFS）约18.9个月^[2]^。对于无驱动突变的非鳞癌，一线免疫联合化疗（如帕博利珠单抗+培美曲塞+铂类，KEYNOTE-189研究）mPFS约9个月^[3]^；鳞癌患者mPFS约6.4个月（KEYNOTE-407研究）^[4]^；PD-L1≥50%的患者一线治疗可选择免疫单药，mPFS约10.3个月（KEYNOTE-024研究）^[5]^。尽管目前一线治疗可显著延长患者生存期，但仍不可避免会出现原发性、获得性耐药，其耐药机制包括：肿瘤蛋白*p53*基因突变（tumor protein P53, *TP53*）等共突变、*EGFR *C797S突变、间质上皮转化因子（mesenchymal epithelial transition factor,* MET*）扩增或人表皮生长因子受体2（human epidermal growth factor receptor 2, *HER2*）扩增、RAS/RAF/磷脂酰肌醇-3-激酶（rat sarcoma virus oncogene family/rapidly accelerated fibrosarcoma/phosphatidylinositol 3-kinase, RAS/RAF/PI3K）通路激活、上皮-间质转化（epithelial-mesenchymal transition, EMT）等^[6,7]^。免疫治疗的耐药则涉及抗原呈递障碍、丝氨酸苏氨酸激酶11基因（serine/threonine kinase 11, *STK11*）突变、Kelch样ECH相关蛋白1基因（Kelch-like ECH-associated protein 1,* KEAP1*）突变、Janus激酶/干扰素γ（Janus kinase/interferon-γ, JAK/IFNγ）信号通路缺陷、免疫抑制性肿瘤微环境及替代免疫检查点，如淋巴细胞活化基因3（lymphocyte activation gene-3, *LAG-3*）、T细胞免疫球蛋白和ITIM结构域（T cell immunoreceptor with Ig and ITIM domains,* TIGIT*）上调等^[8]^。一线治疗耐药后，对于驱动基因阴性的患者，通常建议采用以化疗为主的二线治疗方案。而对于驱动基因阳性的患者，则应依据再次分子检测的结果，选择机制相关的靶向治疗或联合用药方案；若检测未发现可作用的靶点，则可转入传统系统治疗。总体而言，晚期NSCLC患者在二线治疗阶段mPFS为3-5个月，中位总生存期（median overall survival, mOS）为8-12个月^[9]^。

在上述背景下，传统靶向治疗和免疫治疗的疗效逐渐接近瓶颈，临床迫切需要能够兼顾精准治疗和持久抗肿瘤作用的新型药物。抗体偶联药物（antibody-drug conjugate, ADC）的出现，为这一困境提供了新思路。ADC药物通过单克隆抗体精准识别肿瘤表面的特异性抗原，将具有细胞毒作用的药物有效递送至靶细胞内部，从而实现“定向杀伤”，并在发挥抗肿瘤效果的同时，减少全身毒副反应^[10]^。

## 1 ADC作用机制

ADC核心结构由三部分构成：针对肿瘤抗原具有特异识别能力的抗体分子、具备高效杀伤能力的细胞毒性药物以及连接两者的化学连接体^[11]^。尽管不同ADC作用机制存在差异，但它们的核心作用路径大致相同：ADC中的抗体特异性识别并结合肿瘤细胞表面的抗原靶点后，通过内吞作用进入胞内，随后在溶酶体中释放细胞毒性药物，进而通过干扰DNA合成或微管功能等途径导致肿瘤细胞凋亡^[10]^。此外，ADC的抗体与肿瘤细胞受体结合后，还可以阻断下游信号通路传导，直接抑制肿瘤细胞的增殖^[12]^。值得注意的是，部分ADC还具备其他抗肿瘤作用，包括旁观者效应和抗肿瘤免疫效应。旁观者效应是指ADC在靶细胞内被内化降解后，释放出具有膜穿透性的细胞毒性分子，并扩散至邻近的非靶细胞，从而扩大杀伤范围^[13]^。旁观者效应还可通过增加肿瘤血管通透性或诱导肿瘤相关成纤维细胞凋亡等方式改变肿瘤微环境，增强药物的整体抗瘤效果^[14]^。在抗肿瘤免疫方面，ADC中的抗体组分可与免疫效应细胞相互作用，从而激活免疫机制，杀伤肿瘤细胞^[15,16]^。上述多种作用机制使得ADC在抗肿瘤治疗中展现出明显的优势和潜力。

## 2 常见靶点及其代表ADC

针对NSCLC的ADC一直是研究热点，其主要研究靶点包括：HER2、滋养层细胞表面抗原2（trophoblast cell surface antigen 2, TROP2）、细胞间质上皮转化因子（cellular-mesenchymal epithelial transition factor, c-Met）、EGFR、人表皮生长因子受体3（human epidermal growth factor receptor 3, HER3）、癌胚抗原相关细胞黏附分子5（carcinoembryonic antigen-related cell adhesion molecule 5, CEACAM5）以及B7同源物3（B7 homolog 3, B7-H3）等^[14]^。其中针对HER2的ADC曲妥珠单抗德鲁替康（Trastuzumab deruxtecan, T-DXd）和针对TROP2的ADC达托霉素德鲁替康（Datopotamab deruxtecan, Dato-DXd），已相继在美国获批上市，靶向TROP2的ADC芦康沙妥珠单抗（Sacituzumab tirumotecan, Sac-TMT）于2025年3月在国内获批上市。以下将系统梳理不同靶点代表性ADC在NSCLC治疗中的临床疗效和安全性特征，并总结于表1中。

**表1:** 代表性ADCs的综合概述

Item	T-DXd	T-DM1	HER3-DXd	Dato-DXd	Sac-TMT	Teliso-v	TUSA	BL-B01D1
Target	HER2	HER2	HER3	TROP2	TROP2	c-MET	CEACAM5	EGFR/HER3
Agent	Trastuzumab deruxtecan	Trastuzumab emtansine	Patritumab deruxtecan	Datopotamab deruxtecan	Sacituzumab tirumotecan	Telisotuzumab vedotin	Tusamitamab ravtansine	BL-B01D1
DAR	8	3.5	8	4	7.4	3	3.8	8
Half-life	≈6 d	≈4 d	≈11 d	≈5 d	5-7 d	≈3 d	5-7 d	≈7 d
Development stage	Phase III, ongoing	Phase II, completed	Phase III, ongoing	Phase III, completed	Phase III, ongoing	Phase III, ongoing	Phase II, completed	Phase III, ongoing
Cytotoxic payload	Topoisomerase I inhibitor (deruxtecan)	Microtubule inhibitor (emtansine)	Topoisomerase I inhibitor (deruxtecan)	Topoisomerase I inhibitor (deruxtecan)	Topoisomerase I inhibitor (KL610023)	Microtubule inhibitor (MMAE)	Microtubule inhibitor (DM4)	Topoisomerase I inhibitor (Ed 04)
Target population (Previously treated locally advanced/metastatic NSCLC)	HER2-mutant non-squamous	HER2-mutation	HER2 mutation or amplification	HER3 overexpression; EGFR-mutation	TROP2 overexpression	TROP2 overexpression; EGFR-mutation	c-Met overexpression; EGFR wild type	CEACAM5 overexpression
Clinical trial (NCT)	NCT04644237	NCT02675829	NCT04619004	NCT04656652	NCT05631262	NCT03539536	NCT02187848	NCT05194982
Participants (n)	152	49	225	299	64	270	37	113
Efficacy outcomes								
ORR (%)	49	51 (36-66)	29.8 (23.9-36.2)	26.4 (21.5-31.8)	34.0 (23.0-47.0)	28.6 (21.7-36.2)	20.3 (12.3-31.7)	67.5 (50.9-81.4)
mPFS (mon)	-	5.0 (3.5-5.9)	5.5 (5.1-5.9)	4.4 (4.2-5.6)	9.3 (7.6-11.4)	5.7 (4.6-6.9)	-	5.6 (3.9-9.7)
mDOR (mon)	16.8 (6.4-NE)	-	6.4 (4.9-7.8)	7.1 (5.6-10.9)	9.6 (7.6-NE)	8.3 (5.6-11.3)	5.6	-
mOS (mon)	-	-	11.9 (11.2-13.1)	12.9 (11.0-13.9)	-	14.5 (9.9-16.6)	-	-
Safety profile								
ILD incidence (%)	7.0	0.0	5.3	6.8	1.0	9.9	16.0	0.8
Grade≥3 ILD (%)	1.4	0.0	1.3	4.0	0.0	3.6	4.0	0.0
TRAEs (any grade, %)	96	-	96	87.5	100	81.4	-	97
Grade≥3 TRAEs (%)	38.6	2	54	25.6	72	27.9	15.2	64
Common adverse events	Neutropenia, anemia, thrombocytopenia	Neutropenia	Throm- bocytopenia, neutropenia	Stomatitis; anemia, neutropenia	Anemia, leukopenia, neutropenia, stomatitis, alopecia	Peripheral neuropathy, edema, fatigue	Corneal disorder, dyspnea, fatigue	Neutropenia, anemia, leukopenia, thrombocytopenia

ADCs: antibody-drug conjugates; HER2: human epidermal growth factor receptor 2; HER3: human epidermal growth factor receptor 3; TROP2: trophoblast cell-surface antigen 2; c-Met: cellular-mesenchymal epithelial transition factor; CEACAM5: carcinoembryonic antigen-related cell adhesion molecule 5; EGFR: epidermal growth factor receptor; DAR: drug-to-antibody ratio; T-DXd: Trastuzumab deruxtecan; T-DM1: Trastuzumab emtansine; Dato-DXd: Datopotamab deruxtecan; Sac-TMT: Sacituzumab tirumotecan; Teliso-v: Telisotuzumab vedotin; TUSA: Tusamitamab ravtansine; BL-B01D1: Bispecific EGFR/HER3 antibody-drug conjugate; NSCLC: non-small cell lung cancer; ORR: objective response rate; mPFS: median progression-free survival; NE: not estimated; mDOR: median duration of response; mOS: median overall survival; DOR: duration of response; TRAEs: treatment-related adverse events; ILD: interstitial lung disease.

### 2.1 HER2

*HER2*基因突变在非鳞状NSCLC中出现的比例约为3%^[17]^，在不同人群中，该突变率存在一定差异。既往研究显示亚洲人群突变发生率普遍高于欧洲及美洲人群（1.4%-6.7% *vs* 1%-3%）^[18]^，而在我国这一比例达4.3%^[19]^。

T-DXd由三部分组成，第一部分是人源化抗HER2单克隆抗体曲妥珠单抗，第二部分为细胞毒性载荷拓扑异构酶I抑制剂-德鲁替康（DXd），第三部分是可酶切的四肽连接子。T-DXd半衰期约为6 d，主要经肝胆系统代谢排泄。T-DXd药物抗体比（drug-to-antibody ratio, DAR）约为8^[20,21]^，显著高于其他HER2靶向ADC，因此在肿瘤细胞内能释放更多有效载药成分，从而增强对肿瘤细胞的杀伤效应。该药物的连接子可以在溶酶体内被蛋白酶切割，释放的DXd还可通过“旁观者效应”影响邻近HER2低表达癌细胞，从而扩大抗肿瘤范围^[22]^。

II期DESTINY-Lung02研究（NCT04644237）^[23]^验证了T-DXd在经治*HER2*突变型转移性NSCLC患者中的疗效与安全性。该研究设置了两种不同剂量的给药方案：低剂量组为5.4 mg/kg，每三周一次（*q3w*）；高剂量组6.4 mg/kg *q3w*。研究结果显示，高剂量组客观缓解率（objective response rate, ORR）达到56.0%，显著高于低剂量组的49.0%。T-DXd不仅在肿瘤缩小率方面表现突出，而且展现出较长的缓解持续时间（duration of response, DOR），其mDOR可达约16.8个月，有部分患者的病情控制超过12个月。亚组分析进一步指出，药物疗效在既往治疗线数、是否脑转移等因素方面差异不显著，提示T-DXd在*HER2*突变驱动的NSCLC中具有稳定且广泛的抗肿瘤活性。在安全性方面，低剂量组具有优势，尤其是在肺毒性方面表现更好。总体≥3级治疗相关不良反应（treatment-related adverse events, TRAEs）发生率为38.6%（高剂量组发生率为58%），其中中性粒细胞减少（18.8% *vs* 36.0%）和贫血（10.9% *vs* 16.0%）最为常见。间质性肺病（interstitial lung disease, ILD）在低剂量组发生率为7%（其中≥3级为1.4%），而高剂量组为13%（≥3级为2.7%）。综合疗效与安全性评估，5.4 mg/kg *q3w*剂量方案已成为T-DXd在临床上的推荐使用剂量。

为进一步验证T-DXd在中国人群中的疗效与耐受性，开展了DESTINY-Lung05桥接研究（NCT05246514）^[24]^，研究结果显示：中国患者的ORR达58.3%，12个月PFS率为55.1%，与全球研究结果基本一致。安全性方面，≥3级TRAEs发生率为45.83%，主要为血液学毒性与胃肠道AEs。该研究为T-DXd在中国*HER2*突变型NSCLC患者中的注册批准提供了有力依据。

目前，III期DESTINY-Lung04研究（NCT05048797）正在进行中，该研究将比较T-DXd单药（5.4 mg/kg *q3w*）与化疗联合帕博利珠单抗方案在*HER2*突变型转移性NSCLC一线治疗中的疗效与安全性，以明确ADC在该亚型中前移至一线治疗的可行性。若结果积极，T-DXd有望成为首个在*HER2*突变NSCLC一线应用的ADC药物。

恩美曲妥珠单抗（Trastuzumab emtansine, T-DM1）与T-DXd结构类似，是一种由曲妥珠单抗与细胞毒性药物艾坦新（DM1）通过不可裂解硫醚键偶联而成的ADC。半衰期约为4 d，主要经肝胆系统代谢排泄，该药DAR仅为3.5，载药量较低^[25]^。且DM1分子带有正电荷且膜通透性较低，无法从已靶向的肿瘤细胞中扩散至周围HER2阴性细胞，因此T-DM1不具备旁观者效应，在NSCLC中整体疗效不如T-DXd^[26]^。一项单中心II期试验（NCT02675829）^[27]^的NSCLC队列共纳入49例*HER2*突变或扩增的晚期NSCLC患者，该研究结果显示，T-DM1治疗组患者ORR为51%，mPFS为5个月；该试验中T-DM1安全性良好，≥3级TRAEs发生率仅为2%（1/49），最常见的AEs是中性粒细胞减少症，未报告ILD（发生率为0%）。然而，早期有试验^[28]^曾纳入HER2过表达的NSCLC患者进行该药疗效评估，却发现治疗效果并不理想（仅获得ORR 20%）。T-DM1在*HER2*突变型NSCLC中的疗效虽较为显著，但在HER2过表达患者群体中效果受限，这可能是由于肿瘤内外异质性显著，导致仅凭免疫组化（immunohistochemistry, IHC）筛选到的“过表达”人群疗效不稳。

### 2.2 HER3

既往证据^[29,30]^显示，85%-100%的*EGFR*突变阳性NSCLC患者存在HER3过表达。HER3的异常表达不仅参与肿瘤的转移与复发，还被认为与EGFR-酪氨酸激酶抑制剂（tyrosine kinase inhibitors, TKIs）耐药的发生密切相关^[30-32]^。

帕瑞妥珠单抗德鲁替康（Patritumab deruxtecan, HER3-DXd）由人源化HER3靶向IgG1单克隆抗体、DXd及可裂解型四肽连接子组成^[33]^，具有典型单抗的长半衰期特性，半衰期约为11 d，DAR约为8。由于HER3在多数NSCLC患者中呈现普遍高表达状态，因此较其他ADC HER3-DXd具有更广泛的潜在适用人群。HERTHENA-Lung01 II期临床研究（NCT04619004）^[34]^评估了HER3-DXd在既往接受EGFR-TKIs及铂类化疗后疾病进展的晚期*EGFR*突变型NSCLC患者中的疗效与安全性。结果显示，该药ORR达到29.8%，mPFS为5.5个月，mDOR为6.4个月，mOS为11.9个月。进一步的亚组分析表明，HER3-DXd的临床疗效与EGFR耐药机制类型密切相关：在同时存在*EGFR*依赖性和非依赖性耐药机制的患者疗效最佳，ORR为37.5%，其次为*EGFR*依赖性耐药机制患者，ORR为32.4%，而在*EGFR*非依赖性耐药机制患者中疗效相对较差，ORR仅27.2%。在安全性方面，总体人群任何级别TRAEs发生率为96%，≥3级TRAEs发生率为54%，其中血液系统AEs最为常见（发生率>50%），ILD发生率为5.3%。其中≥3级ILD共3例（1.3%），并有1例因ILD导致死亡（0.4%）。目前HER3-DXd与化疗疗效对比的III期临床试验HERTHENA-Lung02（NCT05338970）正在进行中，其结果有望进一步验证药物的临床价值。此外，前期实验研究^[35]^发现，EGFR-TKIs可上调细胞膜HER3表达水平，并促进HER3-DXd的内化和细胞内活性，这一机制为EGFR-TKIs联合HER3-DXd应用于未经治疗的*EGFR*突变型NSCLC患者提供了理论依据。目前，奥希替尼联合HER3-DXd的I期临床试验（NCT04676477）也正在开展中，若获得积极结果，ADC药物与靶向治疗的联合方案有望成为*EGFR*突变型NSCLC的一线选择。

### 2.3 TROP-2

TROP2在多种上皮恶性肿瘤中广泛表达^[36]^，64%-75%的NSCLC患者TROP2呈现高表达状态^[37]^。

Dato-DXd主要由人源化抗TROP2单克隆抗体与DXd共价结合而成^[31]^。该药物半衰期约为5 d，主要经肝脏代谢，DAR为4。III期临床研究TROPION-Lung01（NCT04656652）^[38]^评估了Dato-DXd（6.0 mg/kg *q3w*）与多西他赛（75 mg/m^2^ *q3w*）在经治局部晚期/转移性NSCLC患者中的疗效与安全性，结果显示，Dato-DXd组与多西他赛组ORR分别为26.4%和12.8%，mPFS分别为4.4和3.7个月，mDOR分别为7.1和5.6个月，mOS分别为12.9和11.8个月。PFS获益主要集中在非鳞癌患者中，mPFS为5.5个月，而在鳞癌患者中Dato-DXd未显示出治疗优势，mPFS仅为2.8个月。这一结果在ICARUS-Lung01研究^[39]^中也得到了验证。此外，亚组分析结果显示，在84例伴有脑转移的非鳞状NSCLC患者中，Dato-DXd具有显著的颅内抗肿瘤活性：Dato-DXd组的中枢神经系统ORR为15.8%，多西他赛组为0%。该结果为Dato-DXd在脑转移患者中的应用提供了有力支持。Dato-Dxd治疗组任何级别TRAEs发生率为87.5%（多西他赛组为86.9%），其中≥3级TRAEs发生率为25.6%（多西赛多组为42.1%），最常见的TRAE为口腔炎（55.2%）。与其他ADC相比Dato-DXd血液毒性较低，尤其是中性粒细胞减少症和白细胞减少症发生率明显降低，分别为4.7%和3.0%。但需要注意该药物的肺毒性：在Dato-DXd组中，ILD的发生率约为6.8%，4%的患者出现了≥3级治疗相关ILD，7例出现5级ILD事件。

芦康沙妥珠单抗（Sacituzumab tirumotecan, Sac-TMT）是一种新型TROP2导向ADC。Sac-TMT半衰期为5-7 d，DAR为7.4^[40]^。与Dato-DXd相比，Sac-TMT采用酸碱敏感的可裂解连接子，可在肿瘤微环境（呈酸性）中特异性裂解，精准释放载荷药物（新型拓扑异构酶I抑制剂KL610023），既增强其稳定性和抗肿瘤活性，又减少肿瘤外毒性^[41]^。II期临床研究SKB264-II-08（NCT05631262）^[42]^评估了Sac-TMT在既往接受过治疗、*EGFR*突变的晚期NSCLC患者中的疗效和安全性，结果显示64例患者接受Sac-TMT治疗后，ORR为34%，mPFS为9.3个月。≥3级TRAEs发生率为72%，其中血液毒性最为常见，包括中性粒细胞减少（42%）、白细胞减少（19%）等，ILD发生率较低（发生率为1%），未报告≥3级ILD事件。OptiTROP-Lung03（NCT05631262）研究^[43]^进一步在137例EGFR-TKIs和铂类化疗后进展的晚期*EGFR*突变NSCLC患者中对比Sac-TMT（5 mg/kg *q2w*）和多西他赛（75 mg/m^2^）疗效，结果显示Sac-TMT疗效更佳，ORR为45.1% *vs* 15.6%，mPFS为6.9 *vs *2.8个月。基于Sac-TMT较好的单药活性和可控的耐受性，中国国家药品监督管理局已于2025年3月4日批准芦康沙妥珠单抗上市，用于治疗经EGFR-TKIs和含铂化疗治疗后进展的*EGFR*基因突变阳性晚期非鳞状NSCLC成人患者，Sac-TMT也成为了全球首个获批肺癌适应证的TROP-2 ADC。

### 2.4 c-Met

c-Met蛋白过表达在NSCLC患者中的发生率为25%-39%，可异常激活肝细胞生长因子（hepatocyte growth factor, HGF）/c-Met信号通路，促进肿瘤细胞增殖和血管生成^[44]^；且*MET*基因改变与EGFR-TKIs耐药密切相关，*MET*基因扩增在第三代EGFR-TKIs耐药机制中占比为15%-19%^[45]^。

替利妥珠单抗维多汀（Telisotuzumab vedotin, Teliso-V）为第一代靶向c-Met的ADC，其核心结构为抗c-Met人源化单克隆抗体和微管蛋白的聚合抑制剂单甲基奥瑞他汀E（Monomethyl auristatin E, MMAE）^[46]^，该药物半衰期约为3 d，主要以肝脏代谢为主，DAR为3^[47,48]^。II期临床试验LUMINOSITY（NCT03539536）^[49]^旨在评估Teliso-V在经治的c-MET过表达（≥25%的肿瘤细胞呈现3+染色强度）局部晚期/转移性NSCLC患者中的疗效和安全性，研究结果显示，Teliso-V疗效与c-Met过表达水平无显著相关性，高表达（≥50%的肿瘤细胞呈现3+染色强度）和中表达（25%-50%的肿瘤细胞呈现3+染色强度）组间疗效差异不明显：c-Met过表达、高表达和中表达患者ORR分别为28.6%、34.6%和22.9%，mPFS分别为5.7、5.5和6.0个月，mDOR分别为8.3、9.0和7.2个月，mOS分别为14.5、14.6和14.2个月。Teliso-V安全性良好，任何级别TRAEs发生率为81.4%，≥3级TRAEs发生率为27.9%。常见的AEs包括外周感觉神经病变（30.2%）、外周性水肿（16.3%）和疲劳（14%）。该实验中总体ILD发生率约为9.9%，≥3级ILD的发生率约为3.6%，5级（死亡）为1.8%。较高的肺毒性风险，除与药物所携带的载荷MMAE的潜在肺部AEs谱相关外，还可能与靶点表达范围有关。c-Met在正常气道上皮与肺泡上皮存在低至中度稳定表达^[50]^，提示可能存在“肿瘤外效应”，从而在杀伤肿瘤细胞的同时，诱发或加重局部肺泡上皮损伤与炎性反应^[51,52]^。目前，Teliso-V正在进行III期临床试验（NCT04928846），该实验将在既往接受治疗的局部晚期/转移性、c-Met蛋白过表达、非鳞状*EGFR*野生型NSCLC患者中系统性比较Teliso-V单药与多西他赛的疗效与安全性。值得注意的是，二代靶向c-Met的ADC替利妥珠单抗阿地祖替康（Telisotuzumab adizutecan, Temab-A）在其I期临床试验^[53]^中对c-Met低表达患者也展现出一定治疗潜力，结果显示，Temab-A在c-Met过表达患者中ORR达60%，在c-Met低表达患者中，ORR仍维持在40%。该药的显著疗效可能源于其细胞毒性药物成分：Temab-A采用新型拓扑异构酶I抑制剂Adizutecan，相比传统MMAE具有更广谱的抗肿瘤活性。两代c-Met ADC的初步疗效对比不仅扩大了c-Met靶向的ADC药物的获益人群，同时也为未来ADC的研发优化提供方向。

### 2.5 CEACAM5

CEACAM5在多种实体肿瘤表面过表达，通过促进细胞迁移刺激肿瘤转移^[54,55]^。在NSCLC中，约20%的患者存在CEACAM5过表达^[56]^。

图萨米妥珠单抗雷伐汀新（Tusamitamab ravtansine, TUSA）由可裂解二硫键连接子将人源化抗CEACAM5单克隆抗体与强效细胞毒性药物美登木素生物碱DM4（HY-12454）共价连接而成^[57]^。该药物半衰期为5-7 d，主要经肝脏代谢，DAR为3.8^[58,59]^。II期临床试验（NCT02187848）^[60]^在经治的局部晚期/转移性NSCLC患者中开展。相关研究^[61]^表明CEACAM5高表达患者的高血清癌胚抗原（carcinoembryonic antigen, CEA）水平与TUSA治疗获得高ORR具有相关性，因此该II期试验优先纳入CEA>5 ng/mL的患者，结果显示CEACAM5表达水平与TUSA疗效呈正相关，CEACAM5高表达组ORR为20.3%，中表达组ORR仅为7.1%。这一发现为该药预测性生物标志物的选择及治疗过程中的疗效监测提供了重要的参考依据。试验中TUSA展现出良好的耐受性，≥3级TRAEs发生率仅为15.2%，以可逆性角膜病变/角膜炎、呼吸困难、乏力较为常见，而血液系统AEs发生率与单纯细胞毒性药物方案相比显著降低，但该实验中ILD总体发生率高达16%，其中4%患者≥3级。这一较高的ILD发生率与该实验纳入患者基线状况有关，其纳入多线治疗的晚期非鳞NSCLC 患者，其中约80%接受过免疫治疗，可能引发免疫相关肺毒性叠加效应^[62]^。TUSA的III期临床试验CRAMEN-LC03（NCT04154956）^[63]^已完成，与多西他赛相比，TUSA单药治疗在OS方面呈现改善趋势（12.8 *vs* 11.5个月），但未能达到PFS主要终点（TUSA组mPFS：5.4个月；多西他赛组mPFS：5.9 个月）。该试验结果提示，即便选定特定靶点表达，ADC的优势也并非必然，所以ADC在晚期NSCLC治疗中的定位仍需进一步探索。

### 2.6 双靶点ADC（bispecific ADC, BsADC）

BsADC通过化学连接子将双特异性抗体与细胞毒性药物偶联。与传统的单特异性抗体的ADC相比，BsADC具有双重优势：双抗能够同时识别两个不同的肿瘤相关靶点，从而更精准靶向肿瘤细胞，显著降低对正常组织的损伤；另一方面，双抗结构可以在单一靶点表达下降时保持药效，从而在一定程度上克服耐药问题；此外，两个靶点的协同结合还能促进药物内吞作用，提高有效载荷进入肿瘤细胞的效率^[64]^。目前处于研发阶段的BsADC主要可分为两类：一类是与两种不同的抗原/靶标结合的双异位ADC，例如靶向EGFR和HER3的BL-B01D1、靶向EGFR和c-Met的AZD9592以及靶向EGFR和MUC1的M1231；另一类是与同一抗原/靶标上的两个不同表位结合的双表位ADC，如靶向HER2/HER2的ZW-49。其中国内研制的EGFR/HER3双抗ADC BL-B01D1已进入III期临床试验，展现出良好的应用前景。

BL-B01D1的核心包括由抗EGFR人源IgG1抗体与两个抗HER3人源单链抗体融合形成的双抗结构、拓扑异构酶I抑制剂Ed 04及四肽可切割连接子^[65]^。该药物半衰期约为7 d，主要经肝脏代谢，DAR为8^[66]^。在临床前研究^[65]^中BL-B01D1表现出优于单一靶点ADC的抗肿瘤活性。该药的首项I期临床研究（NCT05194982）^[66]^，共纳入113例经治的局部晚期/转移性NSCLC患者，结果显示在*EGFR*突变型患者中，ORR达到67.5%，疾病控制率（disease control rate, DCR）为87.5%，mPFS为5.6个月；而在*EGFR*野生型患者中，ORR为40.3%，DCR为87.1%，mPFS为5.4个月。尽管疗效显著，但该药AEs较为突出，任何级别TRAEs的发生率高达97%，其中3级以上TRAEs发生率达到71%。血液系统AEs频发：包括贫血（81%）、白细胞减少（72%）、中性粒细胞减少（67%）和血小板减少（62%）。试验中仅报道了1例ILD。鉴于该药在*EGFR*野生型患者中仍显示出可观的疗效，该药III期试验（NCT06382129）正在招募中，该研究旨在比较BL-B01D1与多西他赛在既往接受过免疫治疗和化疗后仍出现疾病进展的局部晚期或转移性*EGFR*野生型NSCLC患者中的疗效差异，以期改善此类患者预后差、生存期短的临床困境。

## 3 ADC的优化路径与临床应用策略

尽管ADC兼具靶向抗体的特异识别能力与化疗药物的强效杀伤活性，在肿瘤精准治疗中展现出显著潜力，但由于其复杂的结构，导致药效与毒性存在不确定性。临床实践表明，ADC在提高疗效的同时，易引发一系列特异性AEs，包括口腔黏膜炎、眼毒性（如角膜、视网膜损伤）、骨髓抑制、恶心呕吐、肝功能异常等。其中，最受关注的是ILD。特别是以旁观者效应较强的DXd为载荷的ADC，其肺毒性具有潜在致命风险^[67,68]^。此外，ADC的临床应用还面临一系列机制和策略上的挑战：（1）耐药机制复杂：既可能来源于靶抗原下调、抗原异质性增加，也可能与溶酶体加工障碍、连接子裂解效率下降、载荷外排泵（如ABCB1家族）上调有关^[69-71]^；（2）治疗顺序不明确：随着T-DXd、Dato-Dxd和Teliso-V等药物相继获批上市，如何在传统TKIs、免疫治疗和化疗之间选择ADC的最佳应用时机仍无统一共识^[72]^；（3）缺乏预测性生物标志物：目前靶点筛选多依赖免疫组化、下一代测序（next-generation sequencing, NGS）来评估表达水平和突变状态，但缺乏能有效反映药物摄取和代谢过程的功能性标志物，因此无法精准筛选获益人群^[73,74]^。

### 3.1 ADC药物结构优化方向

针对上述问题，研究者从分子设计与临床策略两条路径同时进行优化。在药物结构层面，可从ADC的三大核心组分着手：（1）抗体方面：开发亲和力更高、免疫原性更低、组织穿透力更强的抗体片段（如单链抗体可变区片段、抗原结合片段、纳米抗体），以提高靶向选择性并减少非靶组织暴露^[75]^；（2）连接子方面：优化化学结构使其兼具血浆稳定性和可控裂解性，力争实现“只在肿瘤微环境中释放载荷”，例如采用能被肿瘤特异酶（如组织蛋白酶B、β-葡萄糖醛酸苷酶）切割的“智能”连接子^[76,77]^；（3）载荷方面：开发毒性更低、旁观者效应可调控的新型细胞毒分子（如新型拓扑异构酶I抑制剂、微管破坏剂、免疫刺激型载荷），从而提升治疗窗口并改善安全性^[78,79]^。

### 3.2 临床应用策略优化方向

在临床应用层面，ADC的发展正向精准分层管理与多药联合的系统化策略转变。总体目标在于通过识别获益人群、规范毒性管理及探索联合治疗，实现疗效与安全性的动态平衡。

#### 3.2.1 精准识别获益人群

目前，免疫组化和NGS技术已广泛用于靶抗原表达或突变的检测，多组学精准医学的引入可为ADC的个体化应用提供新的契机。现有的研究^[28,80,81]^趋向于整合基因组学、蛋白质组学及空间转录组学等多维数据，筛选具有预测价值的生物标志物，以识别“真正获益人群”。但上述指标都仅能反映静态特征，难以评估药物摄取和代谢过程。未来的研究将重点构建多维功能性生物标志物体系，以动态反映ADC“递送-内吞-裂解-载荷暴露/作用”全过程。例如可以融合分子成像（如免疫正电子发射断层显像）、循环肿瘤DNA动力学、蛋白组/磷酸化组特征结合空间组学，并基于此形成复合预测模型，以实现更高精度的人群分层与疗效预测^[82]^。

#### 3.2.2 加强毒性反应的预测与管理

ADC的复杂结构使其毒性反应具有多样性和不可预测性，其中以ILD最具临床风险。管理应遵循“早分层、早识别、早干预”的原则^[83]^。在用药前，需要系统评估患者的基础肺病、既往放疗史及合并用药情况，完善高分辨率电子计算机断层扫描（computed tomography, CT）与肺功能检测，并进行风险分层。高风险患者（活动性间质病变或严重纤维化者），大多数共识建议避免使用ADC药物^[83,84]^。治疗过程中应定期监测临床症状与影像学变化，并加强患者教育随访。若发生ILD，应分级管理：1级可通过短期糖皮质激素治疗后，在多学科团队评估下慎重复药；≥2级则需永久停药并进行系统性激素治疗^[84]^。近期研究^[85]^已尝试在ADC治疗周期中，利用循环细胞因子（如白细胞介素-6、肺表面活性蛋白相关糖蛋白-6、肺表面活性蛋白-D）以及基于人工智能的影像特征等，共同预测ILD风险，为ADC治疗全过程毒性管理提供新办法。

#### 3.2.3 探索联合治疗以提升疗效并延缓耐药

联合治疗已成为克服ADC耐药、增强疗效的重要研究方向。ADC与免疫检查点抑制剂、EGFR-TKIs或抗血管生成药物联合可通过改善药物递送和通路互补机制实现协同效应^[86]^。其中，ADC联合免疫和化疗方面，以TROP2为靶点的Dato-DXd作为代表性药物，其TROPION-Lung04（NCT04612751）试验^[87]^分别评估Dato-DXd联合度伐利尤单抗/帕博利珠单抗（二联方案）及添加含铂化疗（三联方案）在一线驱动基因阴性NSCLC中的疗效。早期Ib期结果显示：双联组ORR为50.0%，三联组为76.9%；联合方案未出现新的安全性信号，双联疗法和三联疗法患者中≥3级TRAEs发生率分别为42.1%和71.4%。在所有治疗队列中，有4例ILD，其中包括1例1级事件、2例2级事件以及1例4级事件，未观察到5级ILD事件。

此外，ADC与靶向治疗的联合同样具备生物学合理性。以Teliso-V为例，其与奥希替尼联合治疗的Ib期试验（NCT02099058）^[88]^在*EGFR*突变且c-MET蛋白过表达的EGFR-TKIs耐药患者中取得积极结果：ORR约50.0%，mPFS约7.4个月，≥3级TRAEs发生率约19%。这些研究表明联合策略通过协同机制可以实现疗效叠加并延缓耐药。

综上，ADC在NSCLC治疗领域发展迅速，可为既往靶向治疗、免疫治疗或化疗失败后的晚期NSCLC患者提供进一步分子分型驱动治疗。ADC面临耐药性、脱靶毒性、预测性生物标志物筛选等挑战，目前仅用于晚期NSCLC患者后线治疗，未来的发展需在药物结构优化、精准人群筛选、毒性预测与管理以及联合治疗策略等方面持续突破，以在确保安全性的前提下最大化临床获益，实现从“结构创新”到“临床转化”的系统性升级，为更多NSCLC患者提供更为高效、低毒的治疗策略。
